# MicroRNAs as Appropriate Discriminators in Non-Specific Alpha-Fetoprotein (AFP) Elevation in Testicular Germ Cell Tumor Patients

**DOI:** 10.3390/ncrna6010002

**Published:** 2020-01-01

**Authors:** Anna L. Lembeck, Philip Puchas, Georg Hutterer, Dominik A. Barth, Angelika Terbuch, Thomas Bauernhofer, Martin Pichler

**Affiliations:** 1 Division of Oncology, Medical University of Graz, 8036 Graz, Austria; anna.lena.ress@gmail.com (A.L.L.); philip.puchas@icloud.com (P.P.); dominik.barth@medunigraz.at (D.A.B.); angelika.terbuch@medunigraz.at (A.T.); thomas.bauernhofer@klinikum-graz.at (T.B.); 2 Department of Urology, Medical University of Graz, 8036 Graz, Austria; georg.hutterer@medunigraz.at; 3 Division of Oncology and Research Unit for Non-Coding RNA and Genome Editing, Medical University of Graz, 8036 Graz, Austria

**Keywords:** testicular germ cell tumor, AFP, microRNA

## Abstract

Testicular germ cell tumors (TGCTs) are the most commonly diagnosed malignancies in younger men. The monitoring of disease course and recurrence is supported by traditional tumor markers, including α-fetoprotein (AFP). AFP is physiologically synthesized in the liver and can be detected at increased levels in testicular cancer patients as well as under other benign liver diseases, which have been reported as a misleading cause of interpretation of TGCTs clinical course. A cluster of stem cell-associated microRNAs has been reported to outperform traditional tumor markers in newly diagnosed TGCTs, but the value of these microRNAs to differentiate between specific and unspecific AFP elevations, has never been reported. We report here a patient with chronic hepatitis B and normal liver related blood values presenting with a surgically removed primary TGCT and elevated AFP levels. Clinical staging revealed a suspect retroperitoneal metastatic lymph node together with other risk factors and first line treatment with PEB chemotherapy was administered. During curative treatment significantly rising AFP levels led to the assumption of chemo-resistant disease, mandating the initiation of salvage chemotherapy and surgical removal of the putative lymph node metastases. The AFP levels continuously decreased with the interruption of chemotherapeutic agents, indicating a chemotherapy-induced liver toxicity on the basis of pre-existing liver disease. MiR-371a-3p serum levels were not detectable in serum samples with elevated AFP levels. In conclusion, miR-371a-3p may be a reliable biomarker to differentiate between non-specific AFP elevations in TGCTs patients.

## 1. Introduction

Testicular germ cell tumors (TGCTs) are the most commonly diagnosed malignancies in men aged 15 to 40 years in developed countries, accounting for approximately 1% of all male cancers [1]. Though the incidence rate increases over time, the mortality rate declines due to improved multimodal treatment options within the last few decades [2]. In terms of treatment and prognosis, TGCTs are subdivided into two major groups: Seminoma and non-seminomatous germ cell tumors [3]. In metastatic TGCT, prognosis and therapy depend on the location of metastases and tumor marker levels [4]. Patients are generally stratified and managed according to the International Germ Cell Cancer Consensus Group (IGCCCG) recommendations. For treatment response evaluation a follow-up monitoring of the blood-based tumor markers alpha-fetoprotein (AFP), the β-subunit of human chorionic gonadotropin (β-HCG), and lactate dehydrogenase (LDH) are commonly used. However, these tumor markers are increased in only about 60% of TGCT patients. Moreover, in pure seminoma patients, only β-HCG is increased in about 20% of patients [3,4]. Besides a lack of sensitivity, false positive (“non-specific”) elevations of AFP in patients with non-seminomatous germ cell cancer is possible and can be caused by infectious liver disease, liver cirrhosis, or hepatocellular carcinoma [5,6]. Basically, AFP is synthesized by the liver, the yolk sac and gastrointestinal epithelial cells [7]. Under physiological conditions, AFP synthesis is restricted to the fetal development, whereas in adults, elevated levels are indicative for either a malignant process (TGCTs, hepatic tumors) or chronic liver disease [8,9].

MicroRNAs are small endogenous non-coding RNAs with important intracellular and extracellular functions in cancer and normal cells [10]. MicroRNAs harbor some features, making them ideal biomarkers in body fluids including tissue-specificity, chemical stability, robustness, and established detection methods [11]. In recent years, different clusters of microRNAs (miR-371-373 and miR-302) have been shown to be expressed in TGCTs regardless of patient age, tumor site and subtype [12]. Measurable levels in body fluids are therefore proposed as a potential new diagnostic marker in TGCTs patients [13]. Very recently, a prospective multi-center study conducted in more than 600 TGCTs patients demonstrated a sensitivity of 90.1% and a specificity of 94.0% for microRNA-371a-3p to detect TGCTs in patients [14]. Moreover, this microRNA seems to be useful in detecting recurrence of TGCTs after curative treatment [15]. Based on these promising results, we got interested whether this serum-based microRNA might also be helpful in certain cases of non-specific elevation of AFP levels in the setting of a TGCT history as illustrated in the following case report.

## 2. Case Report

**Figure 1:**
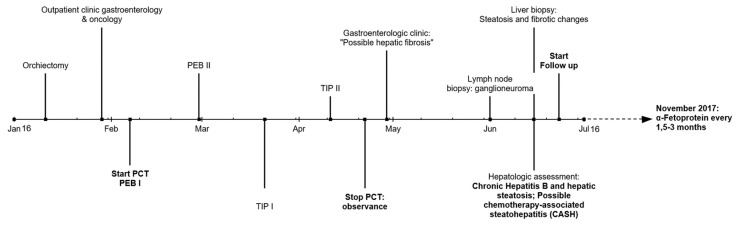
Overview of the treatment timeline including biopsies, clinical re-evaluation and follow up. (PCT = polychemotherapy; PEB = cisplatin, etoposide, and bleomycin; TIP = paclitaxel, ifosfamide and cisplatin).

In this case report, we are discussing the case of a ~45-year-old patient who presented with a swelling of his right testicle. Ultrasound of the right testicle revealed a dense mass and blood tests showed an AFP elevation (365.5 ng/mL, normal range 0–15 ng/mL). β-HCG levels in serum were initially slightly elevated (26.1 U/mL), but remained always in the normal range after surgery (<1.2 mU/mL). LDH levels kept normal during the course of disease. The patient underwent orchiectomy with histological diagnosis of pure embryonal carcinoma (3.5 cm in largest diameter; invasion of blood vessels, AFP-positive). A consecutively performed computed tomography staging described a solitary enlarged retroperitoneal lymph node (1.5 × 0.9 cm) resulting in a clinical stage IIA, good risk according to IGCCCG classification. The patient presented with embryonal carcinoma histology >50%—a generally histological subtype with high risk of relapse in stage I cancer (EAU guidelines 2019)—and vascular invasion in the primary tumor, both criteria warranting the administration of one cycle of PEB chemotherapy even in stage I non-seminoma germ cell tumors. In our stage IIA case, we decided to start with a curative approach with chemotherapy (PEB, cisplatin, etoposide, and bleomycine according to current treatment guidelines (Figure 1)) [4]. 

The patient history revealed that the patient had been a hepatitis B surface (HBs)-antigen-carrier since childhood. Before starting polychemotherapy, the hepatitis-serology showed the following results: HBs-antigen positive, HBs-antibody < 2, HBc-antibody positive. Detection of HBV using PCR was negative. HCV-antibodies and HAV-IgM-antibodies were negative. Regarding the planned cytostatic treatment, a virostatic prophylaxis using Viread (Tenovovir) 245 mg once daily was initiated, as recommended by the consulted hepatologist.

**Figure 2:**
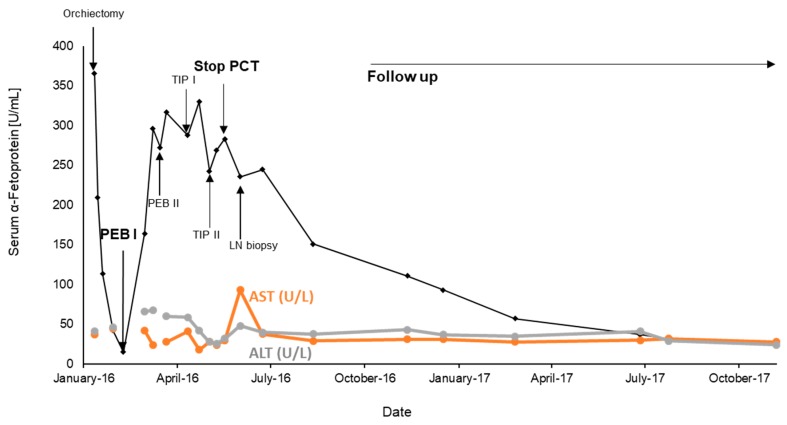
Serum levels of α-Fetoprotein and liver enzymes (AST and ALT) during treatment and further development after finishing of chemotherapy including follow-up (PCT = polychemotherapy; PEB = cisplatin, etoposide, bleomycin; TIP = paclitaxel, ifosfamide, cisplatin; LN = lymph node).

Preoperatively, an elevated AFP level (365.5 ng/mL) was measurable, dropping close to slightly increased values (15.2 ng/mL) after surgery and prior to the initiation of the first cycle of PEB. The serum levels of AFP rapidly increased (Figure 2) under treatment, though radiologic restaging showed no morphologic tumor progression. Based on the observation of rapidly rising AFP levels and a no change course of the putative lymph node metastases in the radiologic follow-up, primary platinum-resistant disease was assumed. Consequently, more intensive chemotherapy using the TIP (paclitaxel, ifosfamide, and cisplatin) regimen was administered. After two cycles of TIP severe polyneuropathy and protracted pancytopenia with grade 3 anemia was observed and required termination of cytostatic treatment. Although the AFP levels were still rising the follow-up CT scans revealed no change in radiological lesions of the lymph node metastasis. Therefore, a more detailed gastroenterological examination of the liver was initiated including a renewed fibro-scan and CT-guided biopsy of the liver as well as removal of the enlarged lymph node. Histological work-up of the liver biopsy revealed advanced liver fibrosis associated with chronic hepatitis B infection and steatohepatitis, consistent with possible chemotherapy-associated steatohepatitis (CASH), whereas the lymph node biopsy revealed ganglio-neurinoma with no signs of metastatic involvement by an embryonal carcinoma anymore. Consequently, antitumor treatment was interrupted, and tightly scheduled follow-up examinations were performed showing a continuous decrease of AFP levels reaching near normal levels one year later (Figure 2). In collected serum samples with elevated AFP values including three independent time points during TIP chemotherapy, we retrospectively measured the levels of miR-371a-3p and miR-367-3p, as well as the frequently used house-keeping miRNAs miR-93-5p and miR30b-5p, by a quantitative RT-PCR-based assay as previously established and reported for a cohort of 52 serum samples of patients with recurrent TGCT disease. The details to the extraction and measurement process are reported previously [15]. The internal house-keeping microRNAs (miR-93 and miR-30b, as described in the methods) were in the same range as in the cohort previously reported (CT-values about 17 to 21). The target microRNA miR-371a-3p was beyond the detection limit (CT-values > 35, whereas in our previous study [15] the CT-values for miR-371a-3p ranged between 23 to 32). Interestingly, though the housekeeping miRNAs were detectable, we could not detect any levels of circulating miR-371a-3p or miR-367-3p (CT-value > 35) in serum samples of this patient. All radiologic follow-up examinations documented no evidence of disease after a follow-up of three years. 

## 3. Discussion

The patient presented here had a medical history of the hepatitis B, a condition known to be potentially associated with increased AFP levels in the blood. Despite anti-viral treatment, AFP levels rose during treatment with curative first-line PEB chemotherapy and again under salvage chemotherapy (TIP). There were no signs of hepatitis reactivation based on liver enzymes or circulating levels of viral DNA. In this patient case, a chemotherapy-associated liver toxicity (as confirmed by the fibro-scan and liver biopsy) probably led to the non-specific increase in AFP levels. These non-specific elevations of AFP in liver diseases have been previously described and may lead to misinterpretation and wrong assumption of tumor progression during curative chemotherapy. MiR-371a-3p has been introduced as a potential novel biomarker in detecting and monitoring primary and metastatic TGCTs [16,17,18]. With this in mind, our previously used and published miR-371a-3p assay could not detect any levels of this novel biomarker in serum samples of this patient [15]. Importantly, we recently reported with the same assay a 13.65 fold higher miR-371a-3p level in patients with disease recurrence in comparison with the same patients without evidence of disease (*p* = 0.014) [15]. In this previously reported study, we observed a 100% sensitivity to detect TGCT with underlying embryonic carcinoma histology. In another study, Dieckmann et al. recently demonstrated in 46 patients that disease relapses had elevated miR-371a-3p levels which subsequently dropped to normal upon remission [14]. In general, miR-371-3p seems to be enriched in germ cells and universally up-regulated in malignant TGCTs where it coordinately downregulates mRNAs involved in biologically significant pathways [19]. Our study is not without limitations. First, the lack of a pre-operative serum samples to confirm the detection of miR-371a-3p in the patient which enables us only to be able to indirectly conclude the high specificity of this biomarker under this condition based on our previous findings [15]. Another limitation is the use of the proper housekeeping gene from serum, as some newer studies also reported issues about pre-analytical influence of haemolysis on the concentration of the serum-based microRNAs [20,21]. In addition, we cannot rule out a lack of expression of this microRNA in the cancer tissue of our patient. First and foremost, the patient became cured and showed disease-free survival of 3 years at the latest follow-up examination. Our case report addresses a rare but important clinical scenario of ruling out unspecific tumor marker elevations by the use of novel biomarkers. Nevertheless, our case report with all its limitations and based on a single case has to be confirmed by larger case series or even prospective studies. In summary, non-specific increased AFP levels are a rare but highly important clinical issue in TGCT patients undergoing curative chemotherapy. Though the evidence that miR-371a-3p can serve as a reliable discriminator between specific and non-specific AFP levels is limited to this case report so far, more and prospective studies are warranted to demonstrate a diagnostic superiority for this novel tumor marker in this clinical scenario. 

## 4. Materials and Methods 

### miRNA Isolation, cDNA Synthesis, and Quantitative RT PCR (qRT-PCR)

For miRNA isolation from serum samples, a miRNeasy Kit (Qiagen, Hilden, Germany) was used to extract total RNA from 200 µL of serum. A three-step procedure was performed to measure miRNA expression in human serum samples. For cDNA synthesis, 50 ng of total RNA were subjected to reverse transcription (RT) using TaqMan microRNA Reverse Transcription Kit (Thermo Fisher, Waltham, MA, USA) and a pool containing four specific 5× RT primers (TaqMan miRNA Assay specific for miR-371a-3p, miR-367-3p, miR-93-5p, and miR30b-5p, Thermo Fisher) following the manufacturer’s protocol, allowing simultaneous reverse transcription of four miRNAs of interest RT was performed on a MyCycler thermocycler (Biorad, Hercules, CA, USA) according to the manufacturer’s recommendations and as previously reported (15). Afterwards, a pre-amplification step was performed using a pre-amp primer pool of four specific 20× TaqMan miRNA assays (TaqMan miRNA assay specific for miR-371a-3p, miR-367-3p, miR-93-5p, and miR30b-5p, Thermo Fisher) and the TaqMan PreAmp Mastermix (Thermo Fisher) following the manufacturer’s instructions. In detail, for each reaction 3.12 µL of RT product were mixed with 6.25 µL of TaqMan PreAmp Mastermix and 3.12 µL of 100-fold diluted pre-amp primer pool. Pre-amp reactions were performed on a MyCycler thermocycler (Biorad). For quantitative RT-PCR pre-amplified PCR products were five-fold diluted using RNAse free water and relative quantification of miRNAs was performed using TaqMan Universal Mastermix II No UNG (Thermo Fisher) and specific 20X TaqMan miRNA Assays on a Light Cycler 480 real-time PCR device (Roche Applied Science, Mannheim, Germany). Expression values were calculated using normalization to miR-93-5p and miR30b-5p [after the formula 2^-(target miRNA-miRNA reference)] and further used for statistical analysis. As an external positive control for the assay, we used a paralleled measured serum pool of samples with radiologically confirmed disease and previous positively tested miR-371a-3p and miR-367-3p samples [15]. Only samples where this serum sample pool gave a positive signal for miR-371a-3p and miR-367-3p (positive means a CT-value below 32), as well as positive signals for the housekeeping miRNAs, were considered as valid. 

As hemolysis can influence serum miRNA levels, we tried to avoid the influence of hemolysis on serum microRNA levels by routinely exploring this hemolysis through the measurement of serum indices (Cobas 6000 system, Roche, Vienna Austria). These serum indices are semi-quantitatively measurements using bichromatic wavelength pairs. Samples with hints to hemolysis (defined by + to +++ positive serum index) were not used for further microRNA analysis. 

The subject gave its informed consent for inclusion before participation in the study. The study was conducted in accordance with the Declaration of Helsinki, and the protocol was approved by the Ethics Committee of the Medical University of Graz (28-383 ex 15/16).

## Abbreviations

TGCT	Testicular germ cell tumors
IGCCCG	International Germ Cell Cancer Consensus Group
AFP	Alpha-fetoprotein
TGCT	Testicular germ cell tumors
LDH	Lactate dehydrogenase
PEB	Cisplatin, etoposide, and bleomycine
PCT	Polychemotherapy
TIP	Paclitaxel, ifosfamide, and cisplatin
HBs	Hepatitis B surface
CASH	Chemotherapy-associated steatohepatitis
